# The Enhanced Durability of AgCu Nanoparticle Coatings for Antibacterial Nonwoven Air Conditioner Filters

**DOI:** 10.3390/molecules28145446

**Published:** 2023-07-16

**Authors:** Fang Zhou, Jiabing Peng, Yujie Tao, Longlai Yang, Dequan Yang, Edward Sacher

**Affiliations:** 1 NanoTeX Lab, Solmont Technology Wuxi Co., Ltd., 228 Linghu Blvd., Tian’an Tech Park, A1-602, Xinwu District, Wuxi 214135, China; 2 Engineering School, Dali University, 2 Hongsheng Rd., Dali 671003, China; 3 Regroupement Québécois de Matériaux de Pointe, Département de Génie Physique, Polytechnique Montréal, Case Postale 6079, Succursale Centre-Ville, Montréal, QC H3C 3A7, Canada

**Keywords:** Ag and AgCu nanoparticles, air conditioner, antibacterial filter, durability, nonwoven

## Abstract

Antibacterial nonwoven fabrics, incorporated with Ag, have been applied as masks and air conditioner filters to prevent the spread of disease from airborne respiratory pathogens. In this work, we present a comparison study of Ag ions: Ag and AgCu nanoparticles (NPs) coated onto nonwoven fabrics intended for use as air conditioner antibacterial filters. We illustrate their color changes and durability running in air conditioners using antibacterial activity testing and X-ray Photoelectron Spectroscopic (XPS) analysis. We found that AgCu NPs showed the best antibacterial efficacy and durability. XPS analysis indicated that the Ag concentration, on both the AgCu and Ag- NP-coated fibers, changed little. On the contrary, the Ag concentration on Ag ion-coated fibers decreased by ~30%, and the coated NPs aggregated over time. The color change in AgCu NP-coated fabric, from yellow to white, is caused by oxide shell formation over the NPs, with nearly 46% oxidized silver. Our results, both from antibacterial evaluation and wind blowing tests, indicate that AgCu NP-coated fibers have higher durability, while Ag ion-coated fibers have little durability in such applications. The enhanced durability of the AgCu NP-coated antibacterial fabrics can be attributed to stronger NP–fiber interactions and greater ion release.

## 1. Introduction

Over the last two decades, the incorporation of Ag-based nanoparticles (NPs) and ions has been widely employed in the textile and health care industries [1,2,3,4,5]. These include cloths, masks [6,7], water disinfection, air conditioner filters [8,9,10,11,12,13], and wound dressing [14,15], used due to their higher antibacterial efficacy and broader spectrum of attack [16,17]. Recently, Ag NP-coated nonwoven fibers have been receiving attention for their protection against COVID-19 when used either as masks [18,19,20,21] or air filters [11,22,23,24].

Apart from the monometallic nanoparticles applied as textile finishing agents, bimetallic NPs have received much interest because of their optical, electrical, magnetic, and catalytic capabilities, and especially their excellent antibacterial properties, which differ dramatically from their monometallic counterparts in most circumstances [25,26]. Bimetallic NPs are made by mixing two distinct metal elements to produce a variety of morphologies and architectures basically synthesized by chemical reduction and biosynthesis in recent studies [27,28]. AgCu NP, as a typical alloy, has been thoroughly studied by us [29,30,31] and others [32,33,34,35,36,37,38] and has been found to possess enhanced antibacterial efficacy, greater than either Ag or Cu NPs, used alone or mixed together [32,39]. This has resulted in reduced cytotoxicity [40] as well.

The durability of an antibacterial fiber is associated with its application requirements (e.g., anti-washing is important for the textiles and filters used in water treatment [41,42], but anti-wind blowing is more important for air conditioner filters). Changing the shape and size of Ag NPs has been used to change the colors of textiles [43,44], although they mostly appear yellow for spherical Ag NP-coated fibers, similarly to Ag NP aqueous dispersions. It is a challenge to determine the color change for textiles treated with Ag ions or NPs [41], although changing the color of the textiles when varying the Ag NP shape is one of the choices [43]. However, the fabrics finished with nano-Ag in different shapes still have the problem of discoloration and degradation, caused over time and by exposure to sunshine [43].

High-Efficacy Particulate (HEPA) filters are extremely efficacious at screening most bacteria and viruses due to their tiny pores (which may be up to 100 nm) [45], but they have high wind resistance (high energy consumption). The most important issue is the risk of the HEPA filters acting as a reservoir for contamination of the indoor air environment [24,46], which means that the filter itself can behave as a source for contamination of the air environment with airborne pathogenic microorganisms if it is not coated with an antibacterial agent [47]. The fabrication of nanofiber nonwovens by electrospinning with the need to fight COVID-19 has recently made significant progress in industrialization. Chinese manufacturers have made nanofiber-filtering nonwovens that can filter viruses, achieve laundering, and be reused for masks [48]. The electrospinning nonwoven filter research progress can be found in a recently reviewed paper [49].

Although Montazer et al. [50] developed a chemical treatment to prevent this, it entails additional costs. While some researchers proposed using a colorless Ag NP-chitosan complex coating [51] for the treatment of fabrics, we found that its color can be changed to yellow at higher temperatures, e.g., 110 °C, or by light exposure in air for one month. Recently, Richardson et al. [52] developed Ag–phenolic (plant polyphenols and antimicrobial Ag ions) coatings on textiles, which can inhibit lipid-enveloped viruses over one thousand times more efficiently than coatings composed of other metal ions, while maintaining their efficacy, even after five washes. Despite this appearing interesting for actual application, its cost may be too high. La et al. [53] recently reported an Ag/graphene-integrated nonwoven polypropylene filter, which is prepared by reducing Ag ions on the surface of graphene nanoplatelets (GNPs) using plant extract. Shiu et al. [54] developed a new filter prepared by Ag@ZIF-8@PP melt-blown nonwoven fabrics with higher air filtration and antibacterial efficacy. Cu is another antimicrobial agent, especially for COVID-19; it has been found that Cu (including CuO) exhibits better performance [55,56,57] than Ag. For example, Perelshtein et al. [58] evaluated a CuO NP-coated nonwoven fabric air filter; their results showed that the CuO NP-coated filter was not only antimicrobial but also detrimental to H1N1 influenza and two SARS-CoV-2 variants. It also demonstrated good stability and mechanical properties. Watson et al. [24] developed a novel antimicrobial treatment for air filters, the approach being to modify an existing filter with a broad-range biocide, chlorhexidine digluconate (CHDG), which is applied to porous filters across the HVAC sector. The advantage is that the filter can quickly kill bacteria and viruses, compared with a metal-based antibacterial coating. Druvari et al. [59] extended this technology and developed a facile and eco-friendly process for the biocidal treatment of commercial high-efficiency particulate air filters for air-cleaning filters. Ag nanowires have also been coated on fabrics as air filters by Park et al. [60]; they found that the overall filtration and antibacterial efficiency of the fibers were significantly improved without affecting the pressure drop.

There are many methods to prepare antibacterial fibers by Ag deposition, which have been extensively summarized in recent review articles [61,62,63], including roll-to-roll coating (economical and extensively used by manufacturers) using Ag NPs or aqueous Ag salts, vacuum sputtering deposition, in situ chemical reduction, etc. They all involve the adhesion of Ag NPs or ions to the fibers, which plays a significant role in their durability. To increase their adhesion, surface modifications of the nonwoven fabric were used, including radiation-induced graft polymerization [64], functionalized amino-terminated hyperbranched polymer ripening [65], chitosan finishing, electron static interaction on protein-coated fibers [66], plasma treatment [67,68,69], etc. For practical applications, water-based NPs dispersion using an appropriate binder is the primary technique, and when using sputtering deposition and plasma, especially air-pressure plasma treatment, combined with roll-to-roll treatment, the NPs coating can be competitive processes for some specific applications.

Although Ag NP coatings have been used for air conditioner filters, there are limited data on their durability. At the same time, our recent studies of AgCu NPs indicated that they showed excellent antibacterial efficacy, which can reduce Ag consumption if they meet the same antibacterial efficacy. Our intention here is focused on evaluating the durability of AgCu NP coatings for nonwoven fabrics as air filters. We also explored their advantages, comparing Ag NPs and Ag ions with PVP-PVA stabilizers, coated onto nonwoven fabric, without using any surface modification processes or special binders. We found that AgCu NP-coated fabrics showed the highest durability, while Ag ion-coated fabrics, with and without PVP-PVA stabilizers, showed the poorest.

## 2. Results

**Figure 1:**
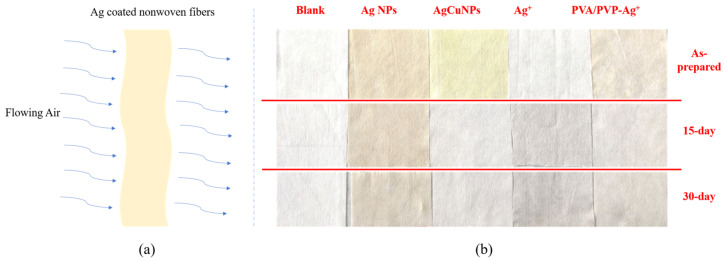
(**a**) Schematic of simulation of Ag-coated nonwoven fiber filters for air conditioning applications; (**b**) Photographs of nonwoven fibers with different Ag coatings and running times.

Firstly, we checked the appearance of the coated nonwoven fabrics as deposited and after running for various periods (0–30 days). After running as depicted in Figure 1a, Figure 1b shows the color change in the various Ag-coated fabrics at different times. After a month, the color of the Ag NPs changed from beige to light brown (Table 1), while that of the AgCu NPs changed from yellow to white. In the case of the fabric coated with Ag ions and PVP-PVA stabilizer, after one month, the color changed from light beige to light gray; without PVA-PVP stabilizer, its color changed from white to light gray.

**Figure 2:**
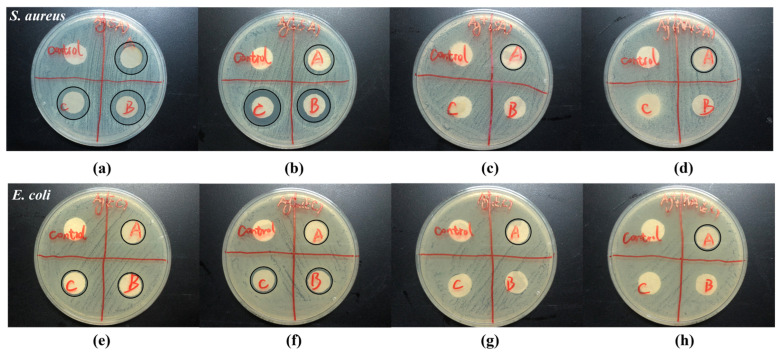
Zone of Inhibition of *S. aureus* and *E. coli* on Ag-nonwoven fibers with different running conditions and treatments. The labeled red letter in the photos indicate the following: A—as prepared; B—running for two weeks; C—running for four weeks. (**a**,**e**) Ag NPs, (**b**,**f**) AgCu NPs, (**c**,**g**) Ag ions; and (**d**,**h**) PVA/PVP-Ag ion-coated samples. The black circles indicate the edges of the inhibition zones.

**Figure 3:**
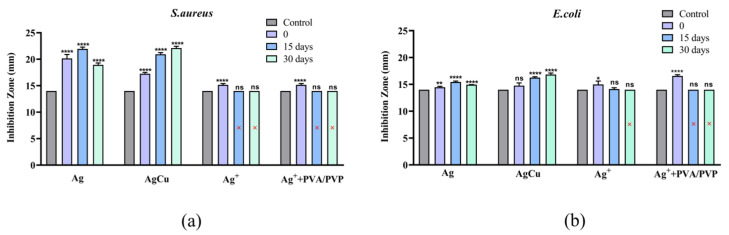
A comparison of antibacterial efficacies of Ag-coated nonwoven fibers by the Zone of Inhibition against (**a**) *S. aureus* and (**b**) *E. coli*. Compared to the control sample, **** denotes a statistical significance of *p* < 0.0001; ** denotes a statistical significance of *p* < 0.01; and * denotes a statistical significance of *p* < 0.05, while ‘ns’ represents *p* > 0.05. *n* = 3. Error bars show standard errors of the mean. The red × indicates that there is no inhibition zone for this sample.

Figure 2 and Figure 3 are antibacterial efficacies determined by the Zone of Inhibition (ZOI) on Ag-coated fabrics under different running conditions. Before this test, we performed a comparison experiment of the ZOI of various pretreatments of fabric samples, shown in Figure S1 in the Supplementary Information. We found that, with a certain amount of deionized water (e.g., fifty μL) to wet the fabric, the antibacterial performance was more obvious and easier to compare; therefore, we used this modification to present the antibacterial activities of the fabric samples. Figure 3a shows that the initial antibacterial efficacies for *S. aureus* are better for both AgCu NPs and Ag NPs compared to Ag ion-coated fibers. Figure 3b shows that there are similar antibacterial efficacies for all samples, except for PVP/PVA and Ag ion-coated fibers, which have better efficacy against *E. coli* bacteria at the initial stage. However, after running for half a month, little antibacterial efficacy remained for the Ag ion-coated fabric, regardless of whether it was coated with PVP-PVA or not for both bacterial strains. For Ag NP-coated fabrics, there was an increase in antibacterial efficacy after two weeks, followed by a decrease after one month of running. In contrast, for the AgCu NP-coated fabric, the antibacterial efficacy increased after running for both a half and a full month. AgCu NP-coated fabric had the best antibacterial efficacy and durability, followed by Ag NPs, while the worst cases were Ag ion-coated fabrics, with or without stabilizers.

**Figure 4:**
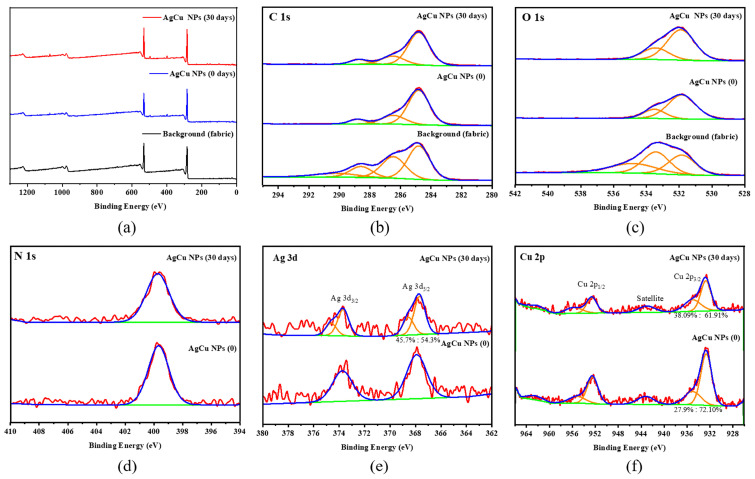
XPS of AgCu NP-coated nonwoven fabrics. (**a**) Survey, (**b**) C1s, (**c**) O1s, (**d**) N1s, (**e**) Ag3d, and (**f**) Cu2p.

**Figure 5:**
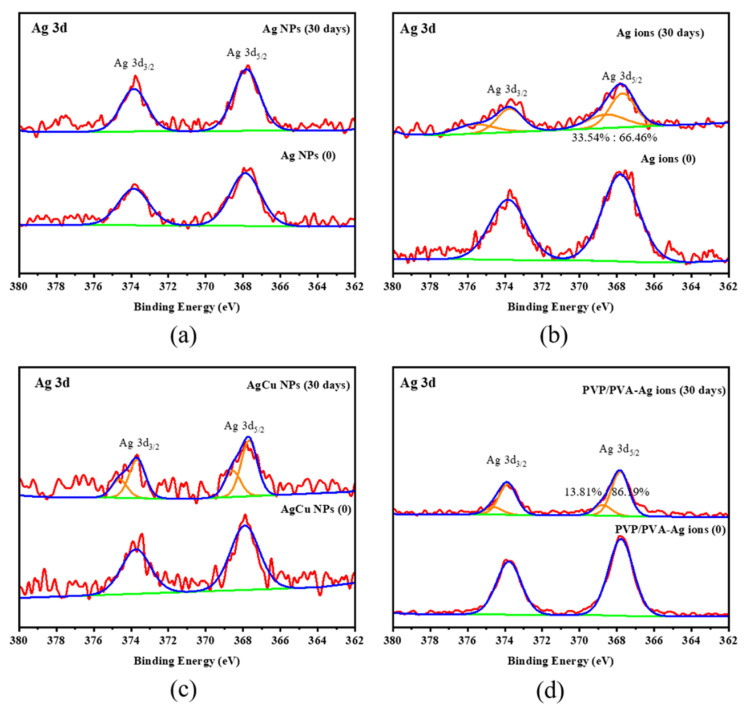
A comparison of Ag3d XPS peak change for the different Ag-coated fibers and running condition of (**a**) Ag, (**b**) Ag ions, (**c**) AgCu, and (**d**) PVP/PVA-Ag ion-coated samples.

Figure 4 shows a survey and high-resolution C1s, O1s, Ag3d, and Cu2p XPS spectra for the AgCu NP-coated fabric. They show the presence of -COOH/C=O (~290/289 eV) and -COH (286.7 eV) peaks in both C1s and O1s, besides the C1s C-C/C-H peak used for energy calibration, consistent with the fabric composition of PE/PP. The Ag3d_5/2_ peak is located at 367.5 eV for the as-prepared sample, while the Ag-O (or -OH) peak appears at 369 eV after running for one month. Cu^2+^ is seen to exist initially, as seen from the presence of the shakeup satellite peak [70], with little change after running for a month. A comparison of Ag3d for the Ag-coated fibers, as shown in Figure 5, indicates that there was some oxidation, except for the pure Ag NP-coated samples, even after running for one month. A higher concentration of the Ag ion-coated fabrics appeared at the initial stage (as deposited, from Table 2) than that of Ag and AgCu NPs, although the same amount of Ag was deposited; this can be caused by the higher surface–volume ratio of Ag ion-coated samples than that of both Ag and AgCu NPs due to larger NPs. This means the smaller the nanoparticles, the stronger the electron emission from NPs [71,72].

Detailed XPS spectra are shown in Figures S2–S4. Chemical compositional changes, estimated using XPS sensitivity factors, are found in Table 2. It is seen that the Ag concentration is ~0.2–0.3% for Ag and AgCu NP-coated fibers and that there was minimal change after running for one month, while that for Ag ion-coated fibers decreased.

These color changes and analytic data (XPS and antibacterial activity) clearly show that the initial color of all coated nonwoven fabrics was changed, especially for the AgCu NP-coated fabrics, which look like uncoated fabrics. Both Ag and AgCu NP-coated fabrics showed no loss of Ag after running for one month, while Ag ion-coated samples, with or without PVP/PVA, showed some loss of Ag.

## 3. Discussion

The color changes in both uncoated and Ag-coated fabrics, induced by air currents in the air conditioner, as shown in Table 1, can be summarized as follows: the color change in the uncoated nonwoven fabric changes from original white to a quite slight gray. This variation is probably due to the deposition of particulate matter during air flow. While the color of Ag NP-coated fabrics, which changes from beige to light brown after running for one month, is principally caused by Ag NP aggregation, which is assisted by air flow. Ag ion-coated fibers, without and with PVP-PVA stabilizers, change from white (or light yellow) to light brown after running for one month, caused by Ag NP aggregation, which we have found previously [73]. This means that no aggregation occurred for the AgCu NP-coated fabrics by air flow, suggesting that the adhesion of AgCu NPs to the fabric is stronger than that of Ag NPs and Ag ions. The initial (as deposited) yellow color of the Ag ion coated with the PVP-PVA sample is due to the reduction in PVP to form nanoparticles [29,30].

Surface chemical analysis by XPS, as shown in Figure 4 and Table 2, indicates that both AgCu- and Ag NP-coated fabrics suffer little loss of NPs, but there is some loss of Ag for the Ag ion-coated fabrics after running for one month. This is further confirmed by the antibacterial data in Figure 2 and Figure 3. The loss of color of AgCu NPs is caused by the formation of an oxidation shell, as confirmed by Ag 3d XPS in Figure 4 and Figure 5, while the antibacterial activity enhances, consistent with Table 1, due to oxidation shell formation [74]. This is because oxidized Ag in AgCu NPs is favorable for Ag ion release in contact with bacteria [75]. However, it is well known that the aggregation of Ag NPs can also result, in some circumstances, in a decrease in antibacterial efficacy [76,77], which may be the main reason for the degradation of Ag NP-coated fabrics.

The XPS results presented in Figure 5 and Table 2 suggest that the adhesion of the Ag ion-coated fabric samples is very weak, leading to the loss of Ag and also NP aggregation on wind blowing. It is well known that fibers, when immersed in solutions of AgNO_3_, in the absence of added reducing agents, undergo a reduction reaction from Ag ions to metallic Ag (Ag^+^ to Ag^0^) [78] due to the presence of functional groups (C=O and C-O) on the fiber surface, as shown by our XPS analysis (Figure 4). The loss of Ag from the Ag ion-coated fabric samples is due to zerovalent Ag having a weak interaction with fibers [79,80], which is a major reason for the loss of Ag from the air current exposure. The antibacterial test data presented in Figure 2 and Figure 3 also confirm Ag loss by air currents for the Ag ion-coated samples.

However, the loss of Ag concentration is minimal for Ag and AgCu NPs, implying that they have a stronger interaction with the fabrics when the wind blows. This enhanced interaction is attributed to the presence of PVP-PVA that can form hydrogen bonds with the fibers [81]. Therefore, the fading of the yellow color of AgCu NP-coated fibers does not affect their application as antibacterial filters, but this partial oxidization process makes a difference in improving the antimicrobial effect, which appears to result from preventing the aggregation of the AgCu NPs.

For the antibacterial efficacy change shown in Figure 2 and Figure 3, it is clearly indicated that both Ag- and AgCu NP-coated fabrics exhibited better antibacterial efficacy for *S. aureus* than for *E. coli*. This is different than the case of Ag and AgCu NPs and Ag ions in aqueous solutions. Secondly, the antibacterial efficacy of Ag NPs is increased in the first 15-day running period, which then decreases after running for one month. The increased antibacterial efficacy of Ag NP-coated fabrics for the first 15 days of airflow may be caused by NP surface oxidation layer formation during that time. The decreased antibacterial efficacy of Ag NP-coated fabrics can be attributed to the Ag NPs aggregation; this is consistent with the Ag NP color change. As one can see in Figure 2 and Figure 3, the antibacterial efficacy of AgCu NP-coated fabric increases for one month, which can be attributed to both surface oxidation and a lack of aggregation. The major reason for the decrease in antibacterial efficacy for the Ag ion-coated fabrics, both with and without PVP-PVA stabilizers during airflow, appears to be a loss of Ag due to a weak interaction of Ag with fibers.

It Is well known that antibacterial efficacy is dependent on the Ag NPs’ size [82,83,84,85], shape, and surface chemistry [86,87,88,89]. The smaller the size, the higher the antibacterial efficacy [90,91] under aqueous environmental conditions. In this work, Ag NPs (12 nm) [29] and AgCu NPs (15 nm; a TEM photomicrograph can be found in Figure S5 in the Supplementary Material) were used. There is no available TEM data for Ag ion-coated fabrics; however, based on the color of the Ag ion-coated fabrics, the average size of Ag may be smaller than 5 nm (without PVA-PVP) and 6–10 nm (with PVA-PVP stabilizers). For the coated fabrics, the antibacterial efficacies, determined from the ZOI diameter against two bacteria, are mainly affected by two factors: Ag and Cu ion release [92,93] and the contact killing mechanism [94,95]. Since the ZOI diameter depends on diffusion, this means that both Ag- and AgCu-coated fabrics have more NPs and ions diffusing than the Ag-coated fabrics, both initially and after running for a month (Figure 3). It appears that Ag ion release plays a more important role in the antibacterial efficacy of the coated fabrics because there is stronger adhesion of the AgCu and Ag NPs to the fabrics, as confirmed by XPS and ZOI testing.

**Figure 6:**
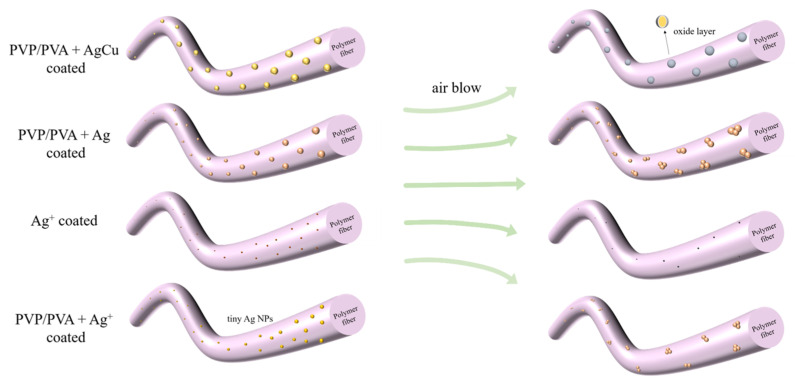
A schematic of Ag-coated fiber colors and property changes on nonwoven fibers from as prepared to running for one month in airflow.

Based on this analysis, a schematic diagram for the coated fiber color and property changes is found in Figure 6. The most notable one is for the AgCu NP-coated samples among these Ag-coated antibacterial fabrics, which not only kept their mechanical durability but also improved their antibacterial efficiency with the moderate oxidation of Ag. It is confirmed by the change to the Ag3d peak, the little change to the Cu2p peak, and the stronger adhesion between antibacterial material and fabric, as determined by ZOI.

Figure 6 highlights the change in the NPs coated on the fabrics. (1) AgCu remains on the fiber, without movement, under air flow, although the surface forms an oxide layer, revealing the stronger interaction between PVP-PVA-capped AgCu NPs and the fibers. (2) The adhesion of Ag NPs on a fiber is likely weaker than that of AgCu since the NPs can move, leading to aggregation. (3) The adhesion of the Ag ions, with and without PVA-PVP stabilizer, is probably very weak, resulting in the loss and aggregation of the Ag NPs on the fibers. The enhanced antibacterial efficacy of the AgCu NPs on the fibers can be due not only to oxidation layer formation to speed up Ag ion release under running conditions but also to Cu enhancing Ag release, which has been found recently [29].

This study provides us with a facile and cost-effective method to maintain stable AgCu NP-based coated antibacterial nonwoven fabric, which can be considered an excellent substitute for colorless antibacterial filters applied in air conditioning to achieve air purification for human health.

## 4. Materials and Methods

### 4.1. Materials

Commercially nonwoven K5310 fabric, composed of polypropylene/polyethylene (PP/PE), was purchased from Jiangsu Beihu New Material Co., Ltd. (Jiangyin, China); Ag and AgCu NP aqueous dispersions were provided by Solmont Technology Wuxi Co., Ltd. (Wuxi, China) at 1000 ppm Ag concentration and PVP-PVA as stabilizers. Ag nitrate (AgNO_3_, 99.8%) was obtained from Tongboxin Hongyin Products Co., Ltd. (Henan, China).

### 4.2. Sample Preparation

*Antibacterial agents*: aqueous dispersions of Ag and AgCu NPs were diluted to 200 ppm Ag concentration, with the Cu concentration at 100 ppm, *w*/*v*, using deionized water. The composite solution of PVP/PVA and Ag ions with 200 ppm Ag ions was prepared by dissolving AgNO_3_ in deionized water with the same amount of PVP and PVA added as in the Ag NP dispersions.

*Nonwoven fabric:* the fabrics were cut to the same size (30 × 33 cm^2^) and soaked in the different antibacterial agents for 1 min before the excess liquid was rolled out and the fabrics were dried by atmospheric exposure. The Ag-coated nonwoven fabrics were dried for 24 h in air at room temperature.

*Air blowing test:* samples of fabric were glued to the air inlet of an air conditioner KFR-35GW/K150+N3, Chigo Air Conditioning Co., Ltd., (Foshan, China) using double-sided adhesive tape. The air conditioner was working continuously for 30 days, and samples were evaluated on days 0, 15, and 30.

### 4.3. Characterization

XPS was conducted on an ESCALab 230i, whose X-ray source was monochromatic Al Kα (1486.7 eV). Survey spectra were conducted with 1.0 eV steps at 100 eV pass energy, while high-resolution spectra were conducted with 0.05 eV steps and a 25-eV pass energy. All spectra were calibrated by placing the C1s peak for C-C/C-H at 284.8 eV.

### 4.4. Antibacterial Evaluations

The antibacterial efficacy of the fabrics was evaluated against Gram-negative *Escherichia coli* (ATCC 8099) and Gram-positive *Staphylococcus aureus* (ATCC 6538). The sub-culture of the bacterial colony was made from 3–5 generations of the primary culture. Bacteria were grown overnight on a nutrient-agar media plate. Inoculums of 0.5 McFarland standards (1.5 × 10^8^ CFU/mL) were maintained in nutrient broth by picking up a single colony from the sub-culture plate [32], and fifty microliters of bacterial solution were added to 5 mL of sterile saline solution to obtain a bacterial suspension at a concentration of 1.5 × 10^6^ CFU/mL for testing. Fabric samples were cut with a 14 mm punch.

Agar dilution is considered to be the gold standard of susceptibility testing or the most accurate way to measure the resistance of bacteria to antibiotics [96]. In this well-known procedure [97], the agar plate surface was inoculated by spreading a volume of the microbial inoculum over the entire agar surface. Then, samples were placed aseptically, using sterile tweezers, onto the surfaces of ager plates. The Petri dishes were then incubated under suitable conditions [98] (37 °C). The antimicrobial agent diffuses into the agar and inhibits germination and growth of the test microorganism, following which the diameters of the inhibition growth zones are measured by a vernier caliper at three or more locations.

### 4.5. Statistical Studies

Statistical data (average ± SD) analyses were conducted, applying One-Way ANOVA (SPSS software Version 8.0 program). This study considered *p* < 0.05 for significantly various groups.

## 5. Conclusions

The durability of directly deposited Ag and AgCu NPs and Ag ions, by dip-roll processes onto nonwoven fabric for air conditioner applications, has been evaluated by antibacterial efficacy, color change, and XPS analysis. We found that the disappearance of the yellow color of AgCu NP-coated fabrics on air current exposure is attributed to the surface oxidation of AgCu NPs without the degeneration of antibacterial activity, while the decreased antibacterial activity and color change for the Ag NP- and Ag ion-coated fabrics can be attributed to surface Ag NP aggregation and Ag loss. PVP-PVA stabilized AgCu NPs, deposited onto the fabric by dip-rolling, appear to be applicable as air conditioning antibacterial filters, leading to higher durability and enhanced antibacterial efficacy. Overall, this study proposes a facile and inexpensive method to maintain stable NP-coated fabrics without using any surface modification processes or special binders but with the improvement of antimicrobial efficacy in use, which may be an effective solution to the implementation of colorless anti-bacterial filters applied in air conditioners to achieve better air purification, particularly for respiratory health.

## Figures and Tables

**Table 1:** Comparison of appearances of Ag-coated fibers after different running times.

Samples	Blank	Ag NPs	AgCu NPs	Ag Ions	Ag Ions-PVA-PVP
As prepared	white	beige	yellow	white	light beige
15 days	white	light brown	white	light gray	light gray
30 days	white	light brown	white	light gray	light gray

**Table 2:** Relative concentration changes in Ag-coated nonwoven fibers by XPS.

	at. %	C1s	O1s	N1s	Ag3d	Cu2p
Sample						
Background (fabric)		74.55	25.45	--	--	--
Ag (0)		75.18	21.77	2.87	0.18	--
Ag (30 days)		71.8	24.19	3.74	0.28	--
AgCu (0)		74.88	21.14	3.38	0.17	0.43
AgCu (30 days)		73.1	22.68	3.53	0.21	0.48
Ag^+^ (0)		75.26	24.38	--	0.37	--
Ag^+^ (30 days)		72.88	26.88	--	0.24 ↓	--
Ag^+^ + PVP/PVA (0)		73.47	25.97	--	0.55	--
Ag^+^ + PVP/PVA (30 days)		75.39	24.22	--	0.39 ↓	--

## Data Availability

Not applicable.

## Supplementary Materials

The supporting information can be downloaded at https://www.mdpi.com/article/10.3390/molecules28145446/s1, Figure S1: A comparison of Zone of Inhibition of *S. aureus* on Ag-nonwoven fabrics with 17 different pretreatments: A, B, and C were wet with 50 microliters of deionized water, while 1, 2, and 3 were not, before 18 antibacterial evaluations, in the order of as prepared (A, 1), two weeks (B, 2) and four weeks (C, 3). Figure S2: XPS of Ag NP-coated nonwoven fabrics. (a) Survey, (b) C1s, (c) O1s, and (d) N1s. Figure S3: XPS of Ag ion-coated nonwoven fabrics. (a) Survey, (b) C1s, and (c) O1s. Figure S4: XPS of PVP/PVA and Ag ion-coated nonwoven fabrics. (a) Survey, (b) C1s, and (c) O1s. (d) N1s. Figure S5: TEM photographs of Ag NPs at (a) low magnification and (b) high magnification; (c) measured particle size; and (d) high-magnification photo of AgCu NPs.
