# Supplementary material for: The Enhanced Durability of AgCu Nanoparticle Coatings for Antibacterial Nonwoven Air Conditioner Filters
Source: Molecules. 2023 Jul 16;28(14):5446. doi: 10.3390/molecules28145446 (PMC10384833; doi:10.3390/molecules28145446)
Supplement: Supplementary file 1 [file molecules-28-05446-s001.zip › molecules-2469563-supplementary.pdf]

S. aureus

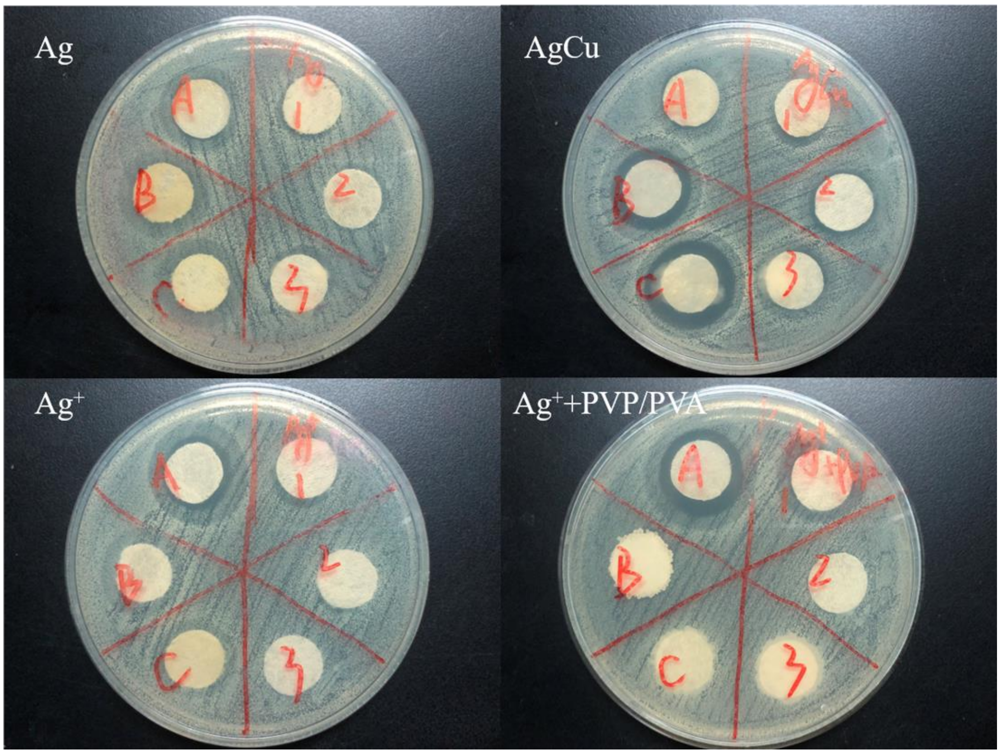

Figure S1. A comparison of Zone of Inhibition of *S. aureus* on Ag-nonwoven fabrics with different pretreatment: A, B, C were wet with 50 microliters of deionized water while 1, 2, 3 were not ,before antibacterial evaluation, in the order of as prepared (A, 1), two-week (B, 2) and four-week (C, 3).

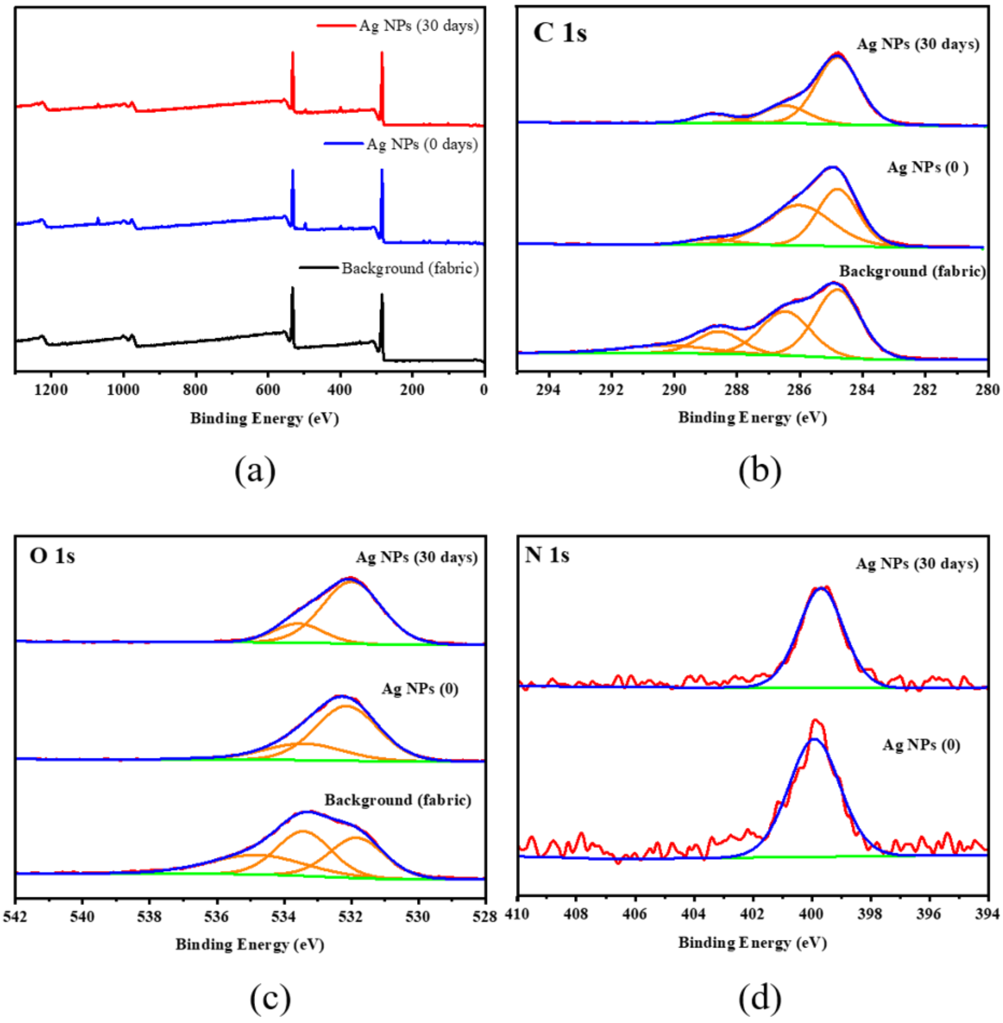

Figure S2. XPS of Ag NPs coated nonwoven fabrics. (**a**) Survey, (**b**) C1s, (**c**) O1s. (**d**) N1s.

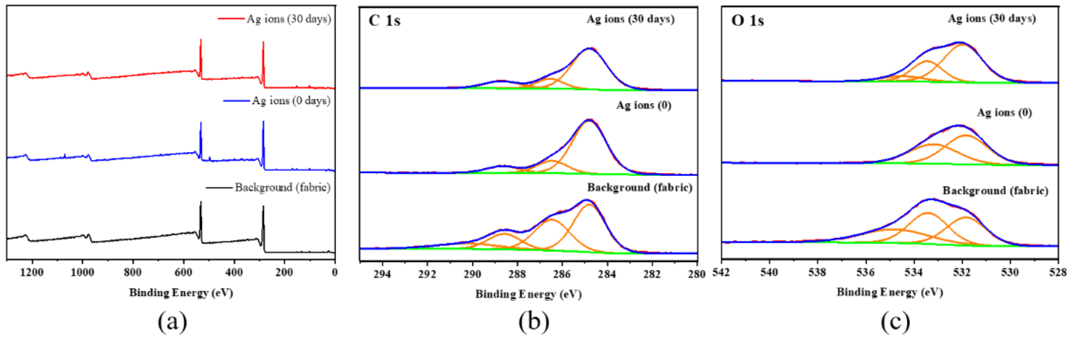

Figure S3. XPS of Ag ion-coated nonwoven fabrics. (**a**) Survey, (**b**) C1s, (**c**) O1s.

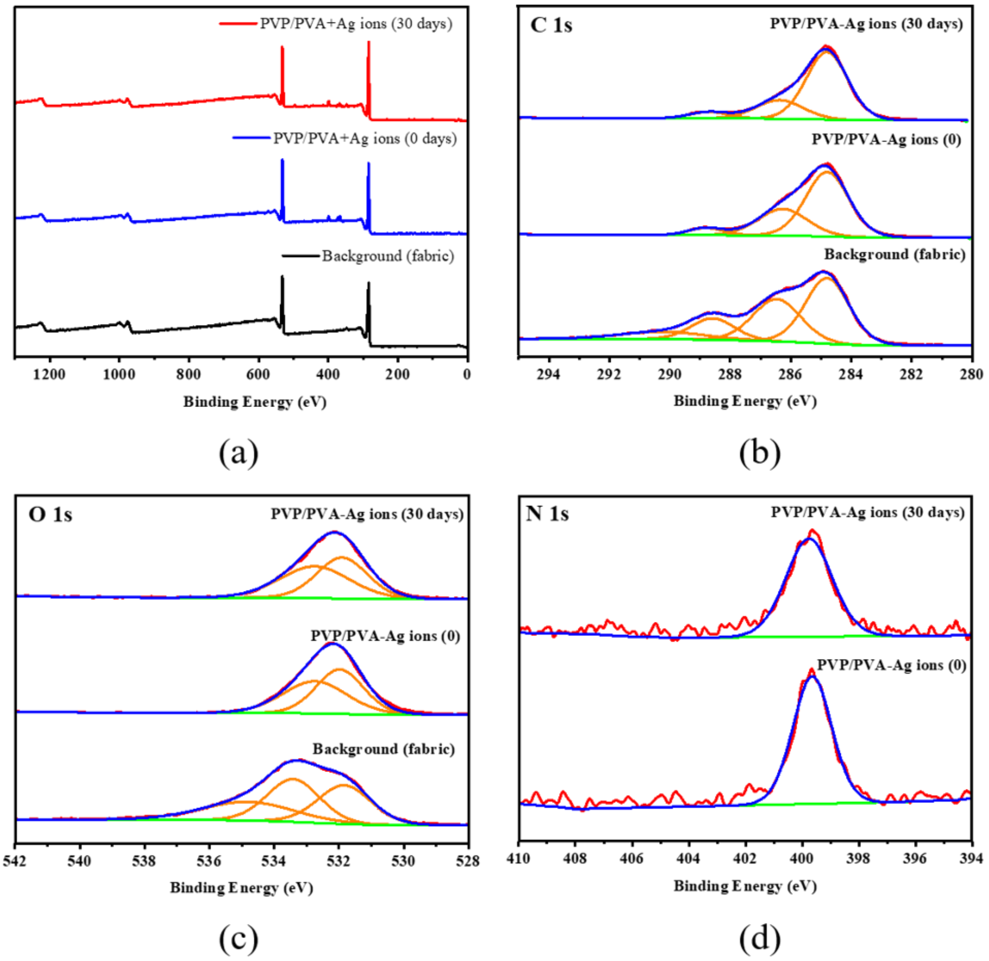

Figure S4. XPS of PVP/PVA + Ag ion-coated nonwoven fabrics. (**a**) Survey, (**b**) C1s, (**c**) O1s. (**d**) N1s.

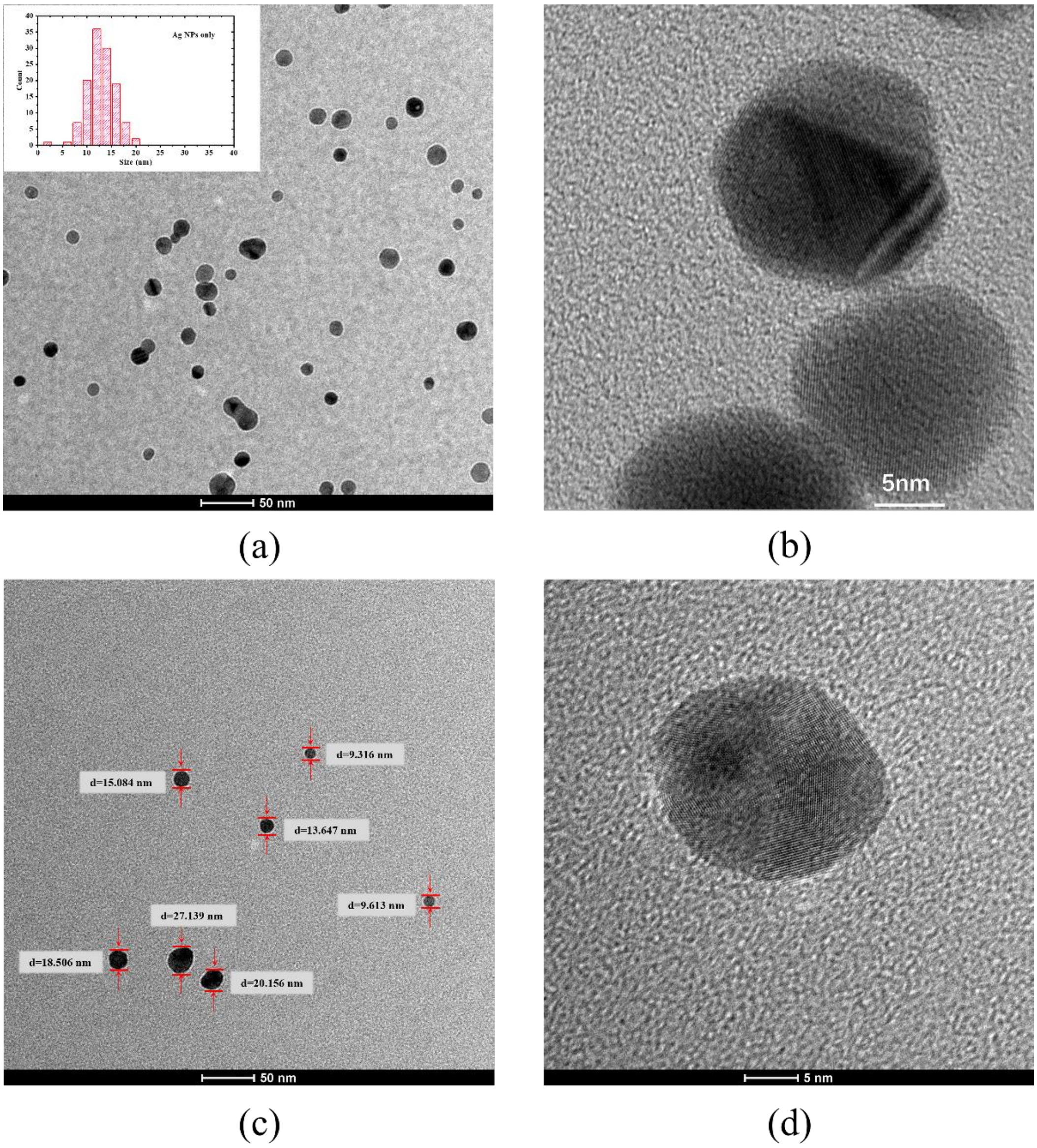

Figure S5. TEM photographs of Ag NPs at (**a**) low magnification, (**b**) high magnification; (**c**) Measured particle size and (d) high-magnification photo of AgCu NPs.
